# Synergistic activity of polarised osteoblasts inside condensations cause their differentiation

**DOI:** 10.1038/srep11838

**Published:** 2015-07-06

**Authors:** Himanshu Kaul, Brian K. Hall, Chris Newby, Yiannis Ventikos

**Affiliations:** 1 Department of Engineering Science, University of Oxford, Oxford, OX1 3PJ, UK; 2 Department of Computer Science, University of Sheffield, Sheffield, S1 4DP, UK; 3 Department of Biology, Dalhousie University, Halifax, Nova Scotia, B3H 4R2, Canada; 4 Department of Infection, Immunity, and Inflammation, University of Leicester, Leicester, LE1 9HN, UK; 5 Department of Mechanical Engineering, University College London, London, WC1E 7JE, UK

## Abstract

Condensation of pre-osteogenic, or pre-chondrogenic, cells is the first of a series of processes that initiate skeletal development. We present a validated, novel, three-dimensional agent-based model of *in vitro* intramembranous osteogenic condensation. The model, informed by system heterogeneity and relying on an interaction-reliant strategy, is shown to be sensitive to ‘rules’ capturing condensation growth and can be employed to track activity of individual cells to observe their macroscopic impact. It, therefore, makes available previously inaccessible data, offering new insights and providing a new context for exploring the emergence, as well as normal and abnormal development, of osteogenic structures. Of the several stages of condensation we investigate osteoblast ‘burial’ within the osteoid they deposit. The mechanisms underlying entrapment – required for osteoblasts to differentiate into osteocytes – remain a matter of conjecture with several hypotheses claiming to capture this important transition. Computational examination of this transition indicates that osteoblasts neither turn off nor slow down their matrix secreting genes – a widely held view; nor do they secrete matrix randomly. The model further reveals that osteoblasts display polarised behaviour to deposit osteoid. This is both an important addition to our understanding of condensation and an important validation of the model’s utility.

The last few years have witnessed a global surge in the number of patients receiving bone defect repairs, with associated costs expected to exceed beyond $5 billion by the year 2020^1^. The rapidly burgeoning field of bone tissue engineering is expected to reduce the bulk of this burden by developing constructs that will enhance bone repair and regeneration^2^. Bone tissue engineering relies on exploiting the principles of bone development, which regulate the *decision-making* and *executive* events observed, initially, during embryogenesis and, later, in the form of regeneration. The template followed on both occasions is very much the same. An essential feature of this template, observed as the decision-making event during osteogenesis as well as development of other mesenchymal structures, is the formation of *condensations*, which are considered as the fundamental unit of morphological change in organogenesis during vertebrate evolution^3^. Condensation is defined as cellular aggregates that result in the formation of more specialised tissues either during embryogenesis and/or regeneration, and is a product of epithelial-mesenchymal interactions. Hall and Miyake^4^ identify condensation as a multi-step process involving initiation, establishment of boundary conditions, cell adhesion, proliferation, growth, and cessation of growth. The foregoing series of events in turn *facilitate regulation of genes*^4^ for either chondrogenesis, which can be replaced by bone (as occurs during *endochondral* ossification where the initially formed cartilage is replaced by bone), or osteogenesis (as occurs during *intramembranous* ossification)^5^ – refer to Fig. 1a. Condensation maturation is, eventually, followed by the differentiation of precursor cells into an osteoblastic lineage, deposition and mineralisation of osteoid, and the terminal differentiation of osteoblasts into osteocytes – collectively, the *executive* event.

Whether endochondral or intramembranous, the dynamics of bone development – especially condensation, its impact on bone deposition, and the subsequent formation of osteocytes – are poorly understood^6^. Thirty five years ago, Knese^7^ hypothesised osteoblast entrapment within osteoid (secreted either by the cell itself causing self-entrapment or by neighbouring cells) as responsible for triggering their differentiation into osteocytes. In the years that followed, Ham and Cormack^8^ suggested that (i) pre-osteoblasts located within condensations deposit osteoid in all directions; Bloom and Fawcett^9^ and Ferretti *et al.* (2002)^10^ opined (ii) non-selective deposition of osteoid due to random orientation of polarised osteoblasts; Romer^11^ and Windle & Nonidez^12^ implicated (iii) synchronised activity of polarised osteoblasts; and Palumbo *et al.* (1990)^13^ and Nefussi *et al.* (1991)^14^ proposed (iv) termination of osteoid deposition as the potential mechanisms responsible for the differentiation of osteoblasts into osteocytes. The aforementioned viewpoints, building further on the entrapment hypothesis – (i) and (ii) implicating self-entrapment, whereas (iii) and (iv) entrapment due to activity of neighbouring cells – are predicated on osteoblast polarity, which in influencing the direction of osteoid deposition regulates osteoblast entrapment. Franz-Odendaal *et al.* (2006)^6^, in the most complete and detailed review on the subject, further elucidated the entrapment hypotheses and outlined, based on the work of the aforementioned investigators, four mechanisms (Fig. 1b), by which osteoblast entrapment can occur. They are as follows:

1. Osteoblasts are apolar and deposit osteoid in all directions. They, therefore, become ‘buried’ in their own osteoid;
2. Osteoblasts can be polarised, and polarity is a property of individual cells. As such, each polarised osteoblast secretes osteoid in one direction only, causing self-entrapment;
3. Multiple osteoblasts can be polarised in the same direction, and polarity is a property of a cellular layer. Therefore, new osteoblast generations (or layers) bury their preceding counterparts; and, finally,
4. Multiple osteoblasts can be polarised in the same direction, but certain osteoblasts in each layer slow down their rate of, or stop, osteoid deposition, thereby undergoing entrapment due to activity of neighbouring osteoblasts.

The manner in which the cells’ organisation^11^,^12^, or lack thereof^9^,^10^, contributes to their (self-) entrapment, and subsequent differentiation, remains unclear. Clarity on the mechanisms of osteogenesis, especially from a quantitative perspective, is crucial to enable the design and development of more robust and optimal bone tissue engineering strategies. As an example, understanding the dynamics governing osteogenesis can help develop more precise culture methods and cellular therapeutics targeted to particular stages of bone development, fracture healing, and correction of segmental defects. Quantified understanding will also assist in determining the optimal number of relevant cells required in a tissue engineered graft to be employed to treat growth deficiencies and/or augment bone density around a foreign implant. Moreover, in quantitating the initiation of mineralisation and osteoblast recruitment, interventions to augment osteogenesis in osteoporosis can be further developed and/or optimised. Furthermore, clarity on the mechanisms of osteogenesis is crucial also, as many skeletal abnormalities and syndromes have their origin in cellular condensations^15^ and deviation from the normal mechanism of osteoid deposition. Finally, a quantitative understanding of condensation, in addition to providing insights into the foremost stage of bone development, will inevitably provide foray into the process of mesenchymal condensation and, thus, mesenchymal organogenesis. In this paper, using intramembranous osteogenesis as an exemplar, we aim to shed light on the developmental dynamics of bone formation.

Observing (one or all of) the hypothesised mechanisms in operation experimentally, however, is almost impossible due to inherent logistical and technical issues. These challenges include the dynamic nature of osteogenesis and the developmental complexity of condensations, which provide the cellular resource from which individual skeletal elements arise. Furthermore, as these hypotheses represent a collage of periodic static snap-shots of osteogenesis, they provide little insight into the initial and boundary conditions required to initiate, observe, and assess the validity of each mechanism. We, therefore, opted for the computational strategy, which is the only available investigative approach for cases such as this, where either suitable experimental techniques/apparatuses do not exist; or experimentation is considered unethical, impossible, or both; or the level of complexity associated with the system under observation makes it difficult to design as well as conduct experimentation. The approach allows for comparison of alternative hypotheses continuously over long periods inexpensively, making them ideal for studying dynamics of biological organisation^16^. As the process of condensation initiation and growth, matrix deposition, and osteoblast differentiation in general, especially as presented in the hypotheses, all have spatial and interactional context to them, agent-based modelling^17^ (refer to Fig. 1c) was employed to (i) simulate the process of condensation formation and osteoid mineralisation, (ii) evaluate the hypotheses in terms of their ability to capture the differentiation of osteoblasts into osteocytes, and (iii) investigate parameters related to osteoblast recruitment and osteoid synthesis that have been implicated in pathological eventualities, such as osteoporosis.

## Results

### 3D agent-based model of *in vitro* osteogenesis

**Figure 2:**
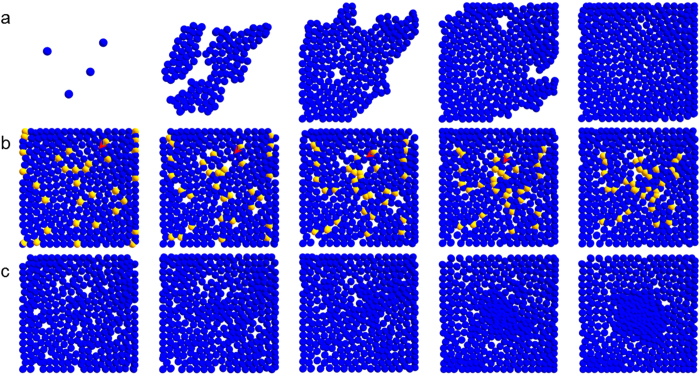
Confluence, migration, and aggregation. The figure shows a series of frames capturing proliferation of the progenitor mesenchymal cells until confluence **(a)** and the migration of pre-osteoblasts to the site of skeletogenesis **(b)**. The largely vacant area begins with five cells that continue to proliferate and fill the entire space. Cells until this time only form a monolayer. They can neither migrate nor grow in the third dimension. The centre of the virtual Petri dish serves as the site of skeletogenesis. Only cells that have acquired a pre-osteoblastic phenotype (orange), unlike the initial fibroblast-like cells (blue), migrate towards the centre. The direction of one of these pre-osteoblasts has been indicated using the red arrow-head. **(c)** displays progression in terms of cellular aggregation. Aggregation can be easily visualised in the final two frames of **(c)**, which show a ventral view of the monolayer after the pre-osteoblastic cells have triggered condensation initiation at the top. As the frames move towards right, time increases by 6.25 days in **(a)**, 30 hours in **(b)**, and 45 hours in **(c)**.

**Figure 3:**
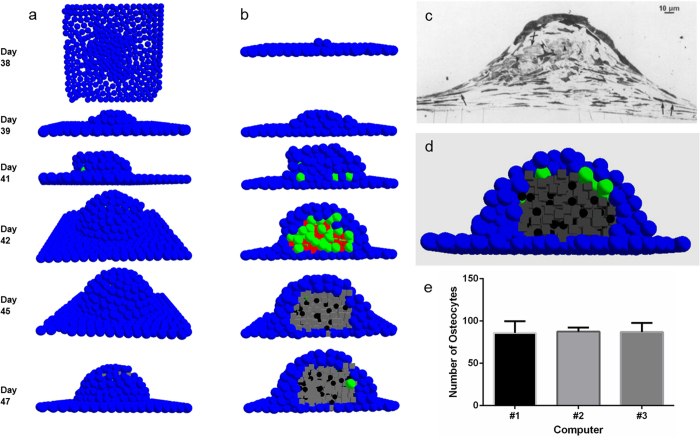
*In virtuo* nodule. The figure displays **(a)** dorsal and **(b)** cross-sectional view of condensation initiation, growth, and mineralisation *in virtuo*. Images in the same row were captured at the same time, also displayed in the figure. As the condensation increases in size, cells in the middle transform into osteoblasts (green) and start depositing matrix (red) instantly. The matrices gradually mineralise (grey) encasing osteoblasts within them, which undergo terminal differentiation into osteocytes (black). **(c,d)** capture the similarities between an *in vitro* developed nodule and its computational counterpart. The basal cells in both cases are fibroblastic and more mesenchymal in nature (blue *in virtuo* and arrows *in vitro*) – they do not participate in bone synthesis. Cells at the top of the nodule, on the other hand, are pre-osteoblastic. The nodules also contain osteoblastic and osteocytic (black *in virtuo* and arrow-heads *in vitro*) cells, the latter embedded within the mineralised matrix (grey *in virtuo* and crossed-arrows *in vitro*). The two nodules differ in their population of osteoblastic cells (higher *in virtuo*), and indicate a difference in their ‘age’. **(e)** shows lack of statistical difference in the number of osteocytes produced by the model(s), when run on three different computers. This was done to ensure that the model was not sensitive to stochastic elements within the code. ***(c)** reproduced with kind permission from Ref.* 20 *© (1988) Elsevier*.

Figures 2 and 3 display the proliferation and spatiotemporal transformation of the precursor (colony forming) mesenchymal cells into a condensation, followed by the deposition and mineralisation of osteoid. These precursor cells proliferate *in virtuo*^18^,^19^ in a cuboidal Petri dish, the geometry and shape of which becomes apparent by the fifth frame in Fig. 2a when cells have completely occupied the entire area. As the cells approach confluence, certain stochastically determined cells differentiate into pre-osteoblasts (shown in orange, Fig. 2b) and begin migrating in a bid to aggregate at the site of condensation. (Whereas *in silico* broadly encompasses numerical models that rely on iterative methods to yield solutions to the underlying ordinary- or partial-differential equations, *in virtuo* – a special case of *in silico* – adds the element of simulating spatiotemporal evolution of a system based on interactions between system components situated within a virtual ‘world’ at each discrete time step).

Although clearly visible only after the third frame in Fig. 2c, the tight aggregation of pre-osteoblasts in the centre of the presented region, the site of osteogenesis, can be observed, in contrast to their neighbouring progenitors. Finally, in Fig. 3a,b, the emergence of a condensation from the site of cell aggregation at various time steps is shown (as observed from the top and as a cross-section). The condensation, initiated by migration, develops due to proliferation of pre-osteoblasts in the third dimension, which continues until the condensation reaches a height of 90 μm. The differentiation of pre-osteoblasts, located in the middle of the condensation, into osteoblasts (green spheres) begins to occur once the condensation has reached a height of 50 μm. Once condensation height increases to 70 μm the osteoblasts start depositing osteoid (red cubes) contributing to further condensation growth as a result of osteoid apposition, which continues until the condensation has reached a height of about 100 μm: commonly attributed as the maximum height achieved by *in vitro* condensations. This is when the deposited osteoid is mineralised (grey cubes). The osteoblasts trapped within the matrix eventually transform into osteocytes (black spheres). Condensation growth stops entirely if the condensation grows beyond 110 μm.

A parallel between the computationally generated *in virtuo* condensation and its *in vitro* counterpart is also presented (Fig. 3c,d). The frame at the top is an electron micrograph montage illustrating the cross-sectional organisation of a mineralising condensation, which developed in an *in vitro* culture of cells derived from 21 day foetal rat calvaria^20^. The cells were treated with ascorbic acid and sodium β-glycerophosphate, which led to the formation of discrete 3D “*nodular structures with the histological and immunohistochemical appearance of a woven bone*”^20^. For detailed information regarding culture method and ultrastructural analyses please refer to the original article. In this frame, cells at the bottom represent the precursor mesenchymal cells (arrows), whereas the top consists of (pre-) osteoblast like cells. Osteocyte-like cells are present within the nodule and are completely surrounded by a dense matrix (arrowheads). Mineralisation in this structure can also be observed (represented by a crossed arrow). The frame below features cross-section of the analogous computational, mineralising condensation possessing similar features. For example, cellular layer at the bottom consists of the precursor cells, while the top consists of pre-osteoblasts. It must be stressed that although both precursor cells and pre-osteoblasts are visualised as blue spheres, they are not treated the same in the model: the former can neither migrate nor proliferate in the third dimension.

The exact moment when a condensation can be categorised as mature is not strictly defined, and could correspond either to the time when osteocytes are first observed within the condensation or, alternatively, when mineralisation is first detected inside the condensation. We opted to work with the former qualification as it is relatively easier to characterise. The time taken for condensation development can vary even within the same organism. Investigators have reported condensation growth and maturation to take anywhere between 3–12 days post-confluence (pc henceforth)^20^,^21^,^22^,^23^. Condensation evolving out of hypothesis #1, #2, and #3 required approximately 9 days pc to achieve maturity, in agreement with empirical observations. Hypothesis #4, however, resulted in condensations that either did not mature at all or required significantly more time to achieve maturation (Kruskal-Wallis test, p < 0.001). By the end of the simulations, osteocytes constituted the major proportion of cells within the virtual condensation, as has been observed experimentally^6^,^24^. Furthermore, hypotheses #1, #2, and #3 during this maturation period produced a mean of 87 osteocytes over the various simulations, which is in agreement with the empirically reported figure for the number of cells within a nodule^6^,^21^,^25^. The agreement between the *in virtuo* nodule structure, condensation maturation time, and the number of osteocytes inside a nodule with *in vitro* data served as *explicit* comparisons, which validated the baseline model.

### Condensation maturation depends upon osteoid synthesis and osteoblast recruitment

**Figure 4:**
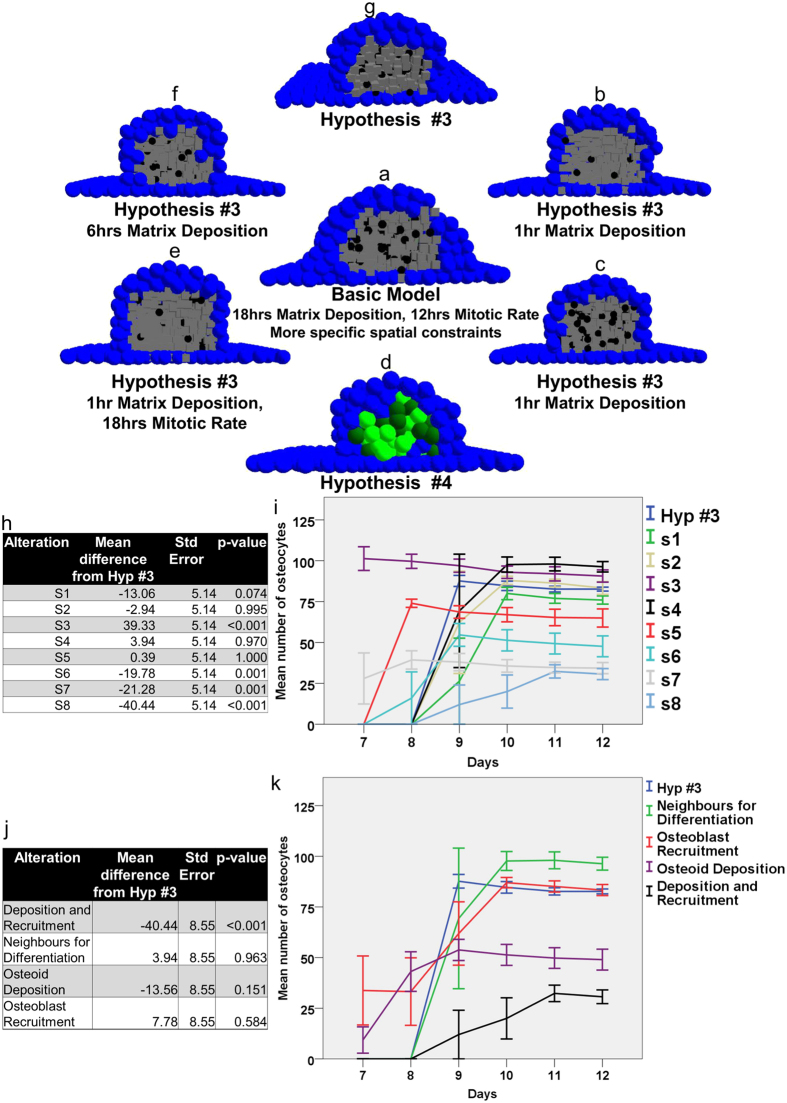
Model sensitivity to parametric alterations. In order to test the basic model’s **(a)** sensitivity to parameters regulating the spatiotemporal development of condensations the following variables were varied: osteoid deposition **(b)**, osteoblast recruitment (in terms of pre-osteoblast proliferation) **(c)**, or both (indicated in the figure) **(d–f)**. More robust boundary conditions pertaining to the spatial aspect of condensation development led to a very realistic condensation structure **(a)**. Extreme increase in the matrix deposition rate of osteoblasts resulted in very few osteocytes being formed **(b,e)**, whereas adding the switching-off gene resulted in stunted condensation growth as can be observed due to absence of osteocytes **(d)**. The images correspond to condensation on day 12 pc. The analysis revealed the dependence of condensation development in the model on two features acting synchronously: osteoid deposition and osteoblast recruitment, which is empirically known. The data presented in tabulated and graphed form demonstrates this quantitatively. In **(h,i)**, comparison of the altered parameters (i.e. matrix deposition rate = 6 hours, etc.) with the hypothesis #3 is presented, whereas **(j,k)** display the comparison of categories (i.e. osteoblast recruitment, etc.) with hypothesis #3. Notice the significant difference only when both osteoblast recruitment and osteoid deposition are co-varied.

Variables pertaining to cellular interactions, which governed condensation development and osteoblast entrapment, were altered to observe how changes might affect the evolving structure. A total of 8 alterations (refer to Table 1) were made from the original model, employing Hypothesis #3 to deposit osteoid (Fig. 4a–g). The alterations can be categorised as affecting *osteoblast recruitment* (*S1*, *S2*, *S3*), *matrix neighbours* required for differentiation (*S4*), *osteoid synthesis* (*S5*, *S6*, *S7*), and both *osteoblast recruitment and osteoid synthesis* (*S8*). The alterations were found significantly different (p < 0.001) using a two-way ANOVA with time as covariate and the alterations as the group variable. When compared individually against the original model (hypothesis #3), employing Dunn’s post hoc test for multiple comparisons, only the following simulations showed significant difference from hypothesis #3: *S3* (p < 0.001), *S6* (p = 0.001), *S7* (p = 0.001), and *S8* (p < 0.001) (Fig. 4h–k). This indicated that the model was generally insensitive to minor stochastic alterations in parameters regulating the spatiotemporal evolution of condensations. For example, employing pre-osteoblast division frequency of 12 (hypothesis #3); 12 ± 3 (*S1*); and 12 ± 1 (*S2*) hours resulted in statistically similar condensations, p > 0.05 for *S1* and *S2* compared with the original. The post hoc comparison to the standard (hypothesis #3), however, suggested that the model is dependent upon osteoid deposition rate as well as osteoblast recruitment. For example, increasing pre-osteoblast proliferation (index of osteoblast recruitment) frequency by 50% (i.e. division every 8 hours instead of 12 hours) led to early formation (p < 0.001) of osteocytes, (day 7 post-confluence compared to day 9 post-confluence from the original case). Similarly, increasing osteoid deposition rate by three times (every 6 hours instead of 18 hours) also increased osteocyte formation rate. Increasing matrix deposition rate by a factor of eighteen, however, even though it led to an increased rate of osteocyte formation (day 7 post-confluence), resulted in significantly few osteocytes in *S7* (p = 0.001). On a similar note, increasing osteoid deposition frequency to every one hour but decreasing proliferation rate by a half (18 hours), *S8*, resulted in delayed condensation maturation, along with the formation of fewer osteocytes. These results have been summarised in Table 1.

The preceding test, however, did not shed much light on the actual process causing the significant difference in the output, as it tested for both *simulation category* and *simulation parameter value*. To evaluate whether the differences observed were attributed to process change only, and not the magnitude of parameter values, we combined simulations that had a similar process change but different magnitude. This led to the four new categories observed: *Deposition and Recruitment*, *Neighbours for Differentiation*, *Osteoid Deposition*, and *Osteoblast Recruitment*, which were subsequently compared with the baseline model. The comparison was made using two-way ANOVA and Dunn’s post hoc test for multiple comparisons, and had greater statistical power as collectively the categories contained more runs than the individual alterations. Only the case where both osteoid synthesis and osteoblast recruitment were altered simultaneously (S8) showed significant difference (p < 0.001). This was an important finding, for it suggested that the development and maturation of the virtual condensation relied on *both* osteoid deposition rate and osteoblast recruitment acting synchronously. This is known to be the case *in vitro* and *in vivo*^6^,^21^,^22^,^23^,^24^,^26^,^27^,^28^,^29^,^30^, where abnormal alterations to either are known to cause pathological eventualities. This is better contextualised by data presented for hypothesis #4 where a minor alteration in osteoid depositing capacity of osteoblasts resulted in abnormal condensation development, even though the pre-osteoblast proliferation rate (or osteoblast recruitment) was the same as for other hypotheses. That the model captures certain governing principles of osteogenesis reasonably accurately is, therefore, quantitatively validated by this analysis.

### Osteoblast polarity dictates the arrangement of osteocytes within condensations

Following basic model development, the strong-inference approach^31^ was used to test the proposed mechanisms. This comparison against the baseline model enabled a meaningful *quantitative* analysis between the various hypotheses and ‘perturbed’ simulations, which would have not been possible due to absence of empirical data that can be explicitly compared across all model output. This is a reasonable strategy because the validated baseline model is but a simplified representation of the (real) physical process/structure that is being simulated. As such, any comparison with the baseline model constitutes an implicit comparison with the physical structure and the validated empirical patterns.

**Figure 5:**
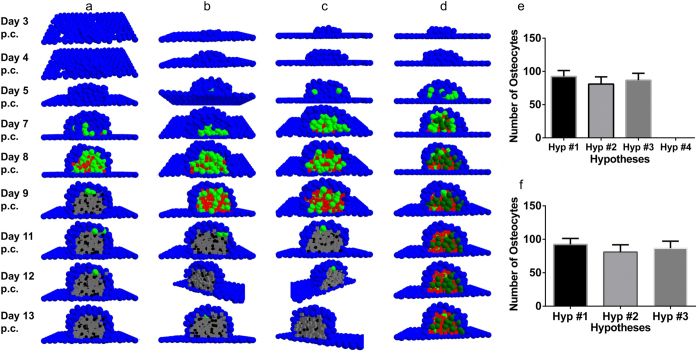
Transformation of osteoblasts to osteocytes according to the proposed hypotheses. The figure shows cross-sectional view of the nodules capturing the transformation of osteoblasts (green) to osteocytes (black). Hypotheses #1–3, displayed by **(a–c)** respectively, roughly yield similar transformation patterns within similar time frames. Hypothesis #4 **(d)**, however, underperforms substantially being unable to either allow the condensation to achieve the right size or osteocytic transformation. This observation is statistically represented in **(e)**, which shows lack of osteocytes in nodules employing hypothesis #4 for condensation development and maturation 12 days post-confluence. In **(f)**, the statistical similarity, as far as the number of osteocytes produced, between hypotheses #1–3 can be observed.

Figure 5a–d displays the process of bone deposition and the transformation of osteoblasts into osteocytes governed by the four hypotheses. Hypotheses #1–3 do not show significant differences between the structure and number of osteoblasts (Kruskal-Wallis, p = 0.095) that form at a given time. Furthermore, the processes of condensation initiation and growth, differentiation of the initial progenitor-cells into osteoblasts, matrix deposition, mineralisation, and osteocyte formation occur approximately at the same time. Hypothesis #4, however, substantially underperforms under this criterion producing, for the dominant majority of simulations, no osteocytes even after 30 days pc (Kruskal-Wallis, p < 0.001). The results were consistent when gathered from simulations conducted across three different work stations (Kruskal-Wallis, p = 0.788). This was done to ensure code insensitivity to random elements within the code, which were used to account for biological stochasticity, and achieved by employing random number generation, which tends to vary between different workstations.

The sequence of events encountered in Fig. 5 is shown without the precursor/pre-osteoblastic cell cover in Supplementary Fig. S1. The inadequacy of hypothesis #4 is further exposed here. However, a more noteworthy observation becomes apparent. The differentiated osteocytes emerging from hypothesis #3 seem well sequestered from each other by the matrix in which they are embedded and are more regularly arranged as compared to hypotheses #1 and #2. In order to quantify this visual observation, a cluster analysis was conducted on data collected for hypotheses #1, #2, and #3. Despite lack of significant structural differences between condensations formed using the three hypotheses, the analysis revealed that hypothesis #3 formed the most consistent number of clusters across iterations, and hypotheses #1 and #2 showed more variation in the number of clusters formed. This (in)consistency correlated well with the observation of visual (ir)regularity in Supplementary Fig. S1. As *clusters* (and their numbers) themselves, are a result and a macroscopic representation of the underlying osteocytes and their arrangement; the cluster and visual analyses were together taken to indicate (ir)regularity of osteocyte arrangement within the nodule. The reasoning being that if the underlying osteocyte arrangement is (ir)regular, the number of resulting clusters will be (in)consistent. Therefore, hypothesis #3 was concluded to result in more ordered and consistent arrangement of osteocytes over iterations compared with hypotheses #1 and #2, which resulted in osteocytes that were irregularly arranged over iterations owing, perhaps, to the varying polarity exhibited by the osteoblasts.

Non-selective osteoid deposition (#1 and #2) was, therefore, observed to cause irregular, non-homogeneous arrangement of osteocytes with certain condensation zones being heavily populated and certain others entirely devoid of osteocytes. This observation deserves a special mention, for not only does it have experimental basis; it also underscores the ability of ABM in capturing emergent behaviour. Ferretti *et al.* (2002)^10^ reported evidence for irregular arrangement of osteocytes that form due to self-entrapment of osteoblasts with arbitrary polarities. In comparison, osteocytes formed due to entrapment from neighbouring cells showed more regular arrangement as well as collective polarity. The model captured both phenomena: self-entrapment represented by hypothesis #1 and #2 resulted in irregular osteocyte arrangement, whereas entrapment represented by hypothesis #3 formed regularly arranged osteocytes. We must emphasise that the resulting observed osteocyte arrangement was neither coded for in the models, nor alluded to as part of initial and/or boundary conditions. It emerged from polarity acquired by osteoblasts dynamically during model run time. This formed an additional piece of validation, for a conjecture based on empirical observation – that self-burial of osteoblasts lead to irregularly arranged osteocytes – was captured by our computational model.

The cluster analysis, furthermore, revealed that hypothesis #2 showed most variation in the number of clusters formed (though it resulted in two clusters more consistently than hypothesis #1). Hypothesis #2 can, thus, be concluded to result in an irregular arrangement of osteocytes, in comparison with hypothesis #1. However, as little is known about the reason underlying irregularity in bone formation, disproving one of the two mechanisms represented by hypotheses #1 and #2 will require more detailed computational analyses. The best fitting number of clusters per iteration is displayed in Supplementary Table S1 for each hypothesis.

### Osteoblasts do not switch-off osteoid deposition as part of normal development

Hypothesis #4, similar to hypothesis #3 in terms of osteoblast polarity, suggested that certain osteoblasts terminate or slow-down osteoid synthesis and end up getting buried by neighbouring osteoblasts. In the initial model, the population fraction that turned-off its ‘genes’ was set to 30%. The period over which termination occurred was indefinite. This combination produced no osteocytes during the periods where other hypotheses resulted in normal condensations. This remained the case when the termination period was decreased to 2 days and 1 day. No differences were observed upon setting the population fraction to 10% (indefinite, 2 days, and 1 day switch-off periods) and 2% (indefinite and 2 days switch-off periods). Only one case from the set of simulations conducted with the 2% and 2 days combination produced osteocytes (Supplementary Fig. S2) but the mechanism failed to capture other events in the same time frame as the other hypotheses; so much so that the differentiation of osteoblasts into osteocytes was not fully observed until the end of simulation. Two problems were identified with this hypothesis: (i) the condensation fails to develop normally in size, which in turn is the cause of many anatomical complications and malformations^32^, and (ii) ‘burial’ of osteoblasts cannot occur properly as there is not enough matrix to properly embed osteoblasts, which, therefore, fail to differentiate into osteocytes. If the deposition of osteoid can indeed be considered *the* most significant reason behind condensation maturation and the differentiation of osteoblasts into osteocytes, hypothesis #4 seems untenable.

Hypothesis #4 only produced osteocytes when, for the population fraction of 2%, the termination period was decreased to 1 day (1.5% of the simulation time). However, this behaviour was critically contingent on the moment when the termination period was applied. If the ‘switch-off’ was imposed too early, a reasonable number of osteocytes resulted. Delayed application of this condition, though, led to inconsistent results. Kruskal-Wallis test conducted on simulations with population fraction and switch off period as 2% and 1 day respectively showed lack of significant difference (p = 0.638) amongst each other (in terms of number of osteocytes observed). While this analysis points to inconsistent performance of hypothesis #4, even when mild limitations regarding population fraction and termination period are imposed, a pattern can be clearly identified. As hypothesis #4 approaches hypothesis #3, i.e. as the effect of population fraction and switch-off period are removed, it starts to produce condensations more consistently. This suggested that hypothesis #4, quite possibly, embodies a pathological departure from hypothesis #3 – a conjecture that required further probing.

### Abnormalities in osteoblast recruitment and osteoid deposition rate are linked to bone-related pathologies

**Figure 6:**
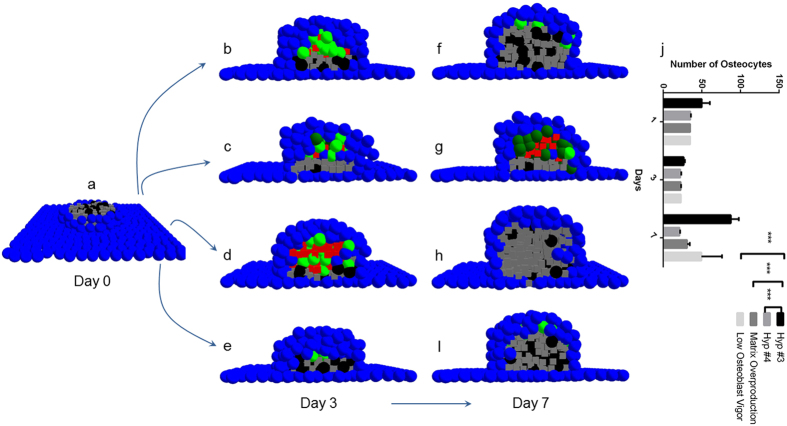
Nodule remodelling following the ‘resorption’ challenge. The figure captures the spatiotemporal development of the condensation following a challenge in the form of bone resorption. As part of the challenge, mineralised matrix of a normal condensation was ‘resorbed’ and the osteocytes necrosed **(a)**. The recovery of this nodule was observed by employing four mechanisms: hypothesis #3 **(b,f)**, hypothesis #4 **(c,g)**, matrix overproduction **(d,h)**, and low osteoblast vigour **(e,i)**. The two frames were taken on days 3 and 7. Nodule remodelling under hypothesis #3 recovered normally producing osteocyte population that was statistically similar to the original, mature condensation. Hypothesis #4 failed to produce a normal condensation in size, osteoid production, as well as osteocyte population. While matrix overproduction did result in a remodelled condensation that resembled the original condensation in size, it had considerably less osteocyte population: a sign of skeletal abnormality. Finally, the condensation that recovered via low osteoblast vigour fared better than the previous two mechanisms, though the amount of osteocytes observed were not statistically similar to the ones observed for the normal, mature condensation, indicating skeletal abnormality. In **(j)**, this information is represented statistically. ***p < 0.001.

Identification of a defect forms the first step towards its correction. Whether *in vitro* or *in silico*, a model of osteogenesis must be dynamic enough to identify a defect and initiate corrective measures. As such, the ability of the model to initiate remodelling and the effectiveness of various hypotheses to govern this remodelling were investigated. Normally developed condensations (via hypothesis #3) were compromised by ‘resorbing’ a significant amount of mineralised osteoid and necrosing a substantial number of osteocytic, osteoblastic, and pre-osteoblastic populations (Fig. 6a). We expected the model to initiate osteoblastic recruitment followed by osteoid synthesis to recreate the condensation. This is exactly what was observed, albeit with the difference that remaining pre-osteoblasts initiated proliferation (index of *osteoblast recruitment* for *in vitro* cases) and differentiated into osteoblasts, which subsequently produced matrix (index of *osteoblast vigour*) that mineralised and aided the differentiation of osteoblasts into osteocytes.

In concurrence with the aforementioned results, hypotheses #1, #2, and #3 resulted in normal condensations, similar to each other (two-way ANOVA for n = 3, p = 0.102). Hypothesis #4, quite obviously, underperformed, resulting in subnormal condensations with sub-optimal amount of matrix and fewer osteocytes, especially in comparison to hypothesis #3 (two-way ANOVA, p < 0.001 for n = 3; Fig. 6b,c,f,g). We attributed this underperformance to low osteoblast vigour. In order to test this conjecture further, hypothesis #3 was modified to (r1) over produce matrix and (r2) suppress osteoblast recruitment and osteoid production (low osteoblast vigour) (n = 3, for both cases), the latter affecting the condensation by depositing less osteoid than needed. Both modifications failed to result in normal condensations (Fig. 6d,e,h,i), two-way ANOVA (p < 0.001), showing a significant deviation from hypothesis #3 (Bonferroni multiple comparison, p < 0.001 for r1, and p = 0.001 for r2; Fig. 6j) but similarity with results obtained using hypothesis #4 (p > 0.05 for both; Fig. 6j). This indicated the mechanism encoded in hypothesis #4 was similar to that obtained using the modified (abnormal) hypothesis #3. It, therefore, seems quite likely that developmental mechanisms represented as hypothesis #4 actually result in pathological bone structures and are, thus, less likely to play any role in normal differentiation of osteoblasts into osteocytes. Furthermore, the investigation also revealed the manner in which matrix overproduction, out of sync with available osteoblast numbers, results in fewer osteocytes due to accelerated condensation growth as a result of enhanced osteoid apposition, which fails to trap enough osteoblasts. While underproduction of osteoid is linked to *osteoporosis*, overproduction is usually associated with *hyperparathyroidism* and *osteitis fibrosa*.

## Discussion

The successful development of computational models is naturally predicated upon adequate validation: i.e. comparison with available “gold standard” datasets. Ideally, this comparison is direct and *explicit*; when such datasets are available. Such direct comparisons are, however, not always possible since the experimental datasets available do not always carry the quantitative information that would make such a comparison possible. In such cases, model validation must occur through a *semi-explicit* strategy where model output is compared with empirical observations and *patterns* – patterns being non-random events.

In order to validate our model we compared maturation time of the virtual condensations with their *in vitro* counterparts. Hypotheses #1, #2, and #3 produced condensations that matured within the empirically observed range of 3 – 12 days pc. Moreover, all normal condensations (refer to the sensitivity analysis conducted on hypothesis #3) matured within this time frame as well. Secondly, the number of cells within a nodule was quantified by Bellows and Aubin^25^ (also discussed by Beresford *et al.*(1993)^21^) to be ~100. Moreover, it is also known that there are about 10 times as many osteocytes as osteoblasts^6^. Considering these two empirical data points, one can conclude that the number of osteocytes within a mineralised nodule should be close to 90. This formed a crucial piece of validation for the model as well as the underlying rules and boundary conditions, for all *normal* condensations (i.e. hypotheses #1, #2, and #3) produced osteocytes −87 ± 11 (mean ± standard deviation) – in agreement with the known empirical figure (especially 90 osteocytes). Simulations that produced ~35 osteocytes were statistically different from the *normal* condensations (as we point in our analyses). Hypothesis #4, though, failed on both accounts. Furthermore, in addition to these two *explicit* comparisons, we presented another *explicit* comparison: *in virtuo* nodule structure in comparison with *in vitro* nodule structure. The spatial arrangement of cells and mineralised matrix, as well as nodule height, reproduced cleanly the *in vitro* observations, which further validates the model as well as the underlying rules.

The model, relying purely on initial and boundary conditions (the latter in terms of spatial limits), presence or absence of neighbours (cells or matrix), instructional relationship between the various agents (cells/matrix), and certain stochastic variables (refer to *Methods*), faithfully reproduced the majority of the template observed during nodule formation as first proposed by Hall and Miyake^32^, as follows.

1. epithelial-mesenchymal interactions;
2. differentiation;
3. condensation formation;
4. deposition of extra-cellular matrix; and
5. terminal differentiation (including mineralisation).

However, we felt that these comparisons though useful were not sufficient and, as such, opted to make additional *semi-explicit* comparisons. These included comparing: (i) factors that regulate condensation development (*in vitro* and *in vivo*); (ii) arrangement of osteocytes within condensations; (iii) impact of osteoblast polarity on osteocyte arrangement; (iv) impact of osteoblast polarity on osteoblast entrapment; and (v) condensation recovery under pathological conditions. Our analyses reveal that (i) both^5^,^20^,^21^,^24^ osteoblast recruitment – captured in the *in vitro* model by pre-osteoblast proliferation – and osteoid deposition must act normally for normal condensation development: failure in even one can result in abnormal condensations (e.g. hypothesis #4 and simulations r1 and r2); (ii) our models produce both irregularly (hypothesis #2) and regularly (hypothesis #3) arranged osteocytes, which can be employed during osteogenesis; (iii) osteoblasts polarised as a layer produce regularly arranged osteocytes whereas osteoblasts with individual polarity give rise to irregularly arranged osteocytes (which happens to be an empirical observation^10^); (iv) osteoblast that act as layers (hypothesis #3) bury neighbouring osteoblasts whereas those that are individually polarised undergo self-burial (hypothesis #2)^10^: our cluster analysis revealed the latter to be producing irregularly organised osteocytes, but was additionally validated by the observation^10^ that those cells that acquire an orientation initially and deposit matrix in that direction throughout their life produce irregular arrangement of osteocytes (we computationally captured that conjecture); and, finally, (v) even normally developed condensations can display poor recovery in case pathological mechanisms (i.e. slow osteoblast vigour/recruitment and abnormal osteoid deposition) become operational during recuperation^24^.

In the biological context, our results refute the view proposed by Palumbo *et al.* (1990)^13^ and Nefussi *et al.* (1991)^14^ that those osteoblasts that switch off their osteoid deposition capacity are transformed into osteocytes. While termination of osteoid deposition will inevitably accompany osteoblast differentiation, a certain case of correlation, it cannot be attributed causality over the event. Furthermore, attributing to a cell this additional event of ‘terminating’ a particular behaviour based on the amount of matrix in its immediate environment is tantamount to adding a layer of complication (rather than complexity) that might be difficult to justify and explain in terms of intra- and extra-cellular features that will additionally come into play if the cells are assumed to display such sensibility and control. The results presented here promote and reinforce Romer’s^11^ as well as Windle & Nonidez’s^12^ view that polarised osteoblasts act synchronously to deposit bone. Contrary to this is the view proposed by Ham & Cormack^8^, Bloom & Fawcett^9^ and Ferretti *et al.* (2002)^10^ that treats acquisition of polarity to be self-regulated. This allows discrete and seemingly random distribution of polarity within a given osteoblast population and, thereby, non-selective deposition of osteoid, which our results indicate lead to clustered and inconsistent osteocyte formation. Our analysis also supports the evidence for this behaviour, reported by Ferretti *et al.* (2002)^10^, suggesting that the direction of osteoid deposition does not alter significantly through an osteoblast lifetime, and rules out Ham & Cormack’s^8^ view that osteoblasts deposit osteoid randomly throughout their lives.

Our analyses, furthermore, provide computational evidence for the view that ossification can employ multiple osteogenic strategies, as reported by Ferretti *et al.* (2002)^10^ who reported both self-burial, hypothesis #2, and entrapment due to neighbouring cell activity, hypothesis #3, as viable mechanisms to deposit bone. The implication is that osteoblasts acquire polarity influenced by their environment rather than by their genomic content alone. The environmental variables can include presence of biological structures or gradients of a chemokine or morphogen, amongst others^33^. Whichever case it may be, the environmental impact will be continuous, and, therefore, it can be argued, most cells within a population would be influenced collectively, rather than individually. For example, if gradient of a particular solute is assumed to contribute to polarity, it is logical to conclude that a collection, or layer, of cells, rather than one cell, would be affected. This layer of cells will then act in alignment to carry out functions influenced by their polarity; such as, deposition of osteoid and, in turn, the burial of neighbouring (arguably, layer of) osteoblasts. This is perhaps the reason why self-burial (hypothesis #2) mainly contributes to the formation of the core of intramembranous bone structures, such as the primary trabeculae, which form around the vascular framework where gradients will be low owing to presence of high solute concentration (hence little impact on polarity); whereas entrapment due to activity of neighbouring osteoblasts (hypothesis #3) seem to be mainly involved in bone compaction, at sites away from the vasculature where solute gradients will become more appreciable.

As the shape and size of cartilage condensations is a predictor of early endochondral bone morphology^4^, understanding the process as well as the regulatory events that guide the structural topography of condensation(s) is of great interest, for alterations can result in skeletal defects^34^. Condensations have, therefore, been termed collectively as the ‘membranous skeleton’ to highlight their existence and equal status with cartilaginous and osseous skeletons^4^,^32^. A significant feature of our investigation remains the fact that our approach enabled us to observe development of abnormal condensations (hypothesis #4), and allowed us to investigate parameters that are linked to bone pathology. Additionally, the model, being inherently dynamic in nature, provided a novel foray into the dynamics of bone remodelling (*sans* osteoid resorption), both normal and pathological. For example, the model suggested both under- and over-production of osteoid (index of osteoblast vigour) as well as low and high pre-osteoblast proliferation (index of osteoblast recruitment) to contribute towards abnormal condensation development. This has implications in studying bone defects such as *osteoporosis*, where the model can be employed to better understand the role of aforementioned parameters in causing low bone density, as well as cases where bone deposition seem to be amplified (e.g. *osteitis fibrosa*). Furthermore, by introducing into the model interactions between a drug molecule and the targeted ‘agent’, usually osteoblasts, the efficacy and impact of the given drug on osteoblast activity or recruitment can be evaluated. The model can, therefore, be used to optimise drug development. The fact that we were able to analyse parameters that caused abnormality and, more importantly, optimise them to lead to normal condensation development constitutes the principal beauty of the agent-based paradigm.

The model relied on cellular migration as the initiator of condensations. *In vivo,* mesenchyme close to the site of skeletogenesis helps furnish condensations with cells, which migrate to the relevant site, as indicated by Jabalee *et al.* (2013)^5^. The conclusions of our model, therefore, with respect to osteoblast differentiation, apply equally to *in vivo* cases, especially considering that osteoid deposition occurs after migration and differentiation of pre-osteoblasts. Similarly, even though the model was coded considering intramembranous ossification the findings from this investigation pertaining to bone remodelling can be easily extended to bone structure formed as a result of endochondral ossification. Furthermore, the model assumed optimal nutrient and morphogen concentrations, which are only justifiable for *in vitro* studies. Similarly, polarity, even though acquired dynamically by osteoblasts during development as well as remodelling in our investigation, had a purely spatial character. *In vivo*, this will not be the case, for the chemical and mechanical environment of osteoblasts will also influence polarity. The two assumptions, however, will not alter our conclusions as we aimed to investigate the performance of the proposed differentiation mechanisms under normal developmental circumstances. Moreover, the agent morphology employed in the model was idealised, though it would not upset our conclusions as agent morphology was not considered to play any role in influencing either osteoblast polarity or its differentiation. Finally, in this investigation, growth and maturation of only one condensation/nodule was considered, but, once again, the conclusions can be extended to *in vitro* cultures with multiple condensations/nodules without any loss of generality.

Besides aiding our fundamental understanding of a significant biological event, results from this investigation display how computations can serve as efficacious supplements to experiments. As the philosophy behind the use of computational strategies is to condense the lab into the computer, and the experiment into the code^35^, we also tested the model’s sensitivity to stochastic variables within the code as well as result reproducibility across various computer systems. This was achieved by running models (employing the four hypotheses) on separate workstations. Simulations resulted in similar condensation structures with similar osteocyte populations within the same time frame. The fact that we reproduced our results on more than one machine means that our model (code available under Creative Commons Attribution License at *www.flame.ac.uk*) as well as results can be reproduced and modified by anyone anywhere, and further used for hypothesis testing of any kind. In particular, this investigation underscores how the discrete mathematical approaches, specifically, agent-based modelling can be applied to recreate experiments that for a variety of reasons may be difficult to design and execute. It must be reiterated that these hypotheses could not have been tested experimentally, especially in the detail presented here. The mechanism(s) governing osteoblast differentiation being unknown, it is immensely challenging, if not outright impossible, to engineer a cellular population that can obey *in vitro*, and most certainly *in vivo*, mechanisms represented by the four hypotheses. Moreover, despite the availability of mutated osteoblasts, which display abnormal osteoid deposition, it must be kept in mind that the experiments evaluating these hypotheses require precursor cells to undergo two degrees of differentiation to form osteoblasts, which can subsequently display abnormal osteoid deposition or randomly acquired polarity. Currently, this can only be achieved *in virtuo*. However, mutated osteoblasts can be employed *in vitro* to quantify the contribution of hypothesis #4 in causing bone related pathologies – an investigation we intend to undertake next.

Furthermore, agent-based modelling, in offering a fresh perspective to investigators interested in exploring the impact of activity of a collection of entities (enzyme, cell, extracellular-matrix, etc.) on global observables, opens up previously inaccessible data. Finally, the fact that we were able to observe differences in condensation maturation that emerged due to the differences in the hypotheses tested as well structural features not explicitly included within the code, points to the robustness of the technique as a hypothesis testing tool. Given that agent-based modelling is the most intuitive way of simulating biological systems, we hope that results from this investigation shall encourage biologists, developmental or otherwise, to pick up this methodology to investigate questions that have proved challenging to explore due to lack of suitable apparatuses and/or techniques, or other logistical or ethical issues.

In conclusion, we present, to the best of our knowledge, the first 3D agent-based model of *in vitro* intramembranous condensation and osteogenesis. The model was reinforced by a range of sensitivity tests and evaluation of model output following a physiologically relevant challenge (bone resorption). Overall, data from more than 125 simulations were analysed. The model, capable of capturing empirically observed emergent behaviour during osteogenesis, suggests that osteoblasts display polarised behaviour and act in synchrony to deposit bone and result in dynamic and static bone formation. The model also revealed osteoblasts do not during normal development switch-off their osteoid deposition genes to trigger their differentiation into osteocytes. In fact, this behaviour was associated with pathological bone development during both bone morphogenesis and regeneration. Our conclusions form a significant conceptual, and technical, advancement compared to the current literature and understanding on this topic and resolve speculations that have been around for a good four decades.

## Methods

### Computational Approach: Background

Agent-based modelling (ABM) is a class of discrete mathematical models that treats a system as a collection of discrete autonomous decision-making entities, known as *agents*^17^, capable of acting at each of various discrete time steps depending upon their local environment and on rule-sets attributed to them^36^. An agent-based model, in addition to the initial and boundary conditions, has three essential elements: agents, environment, and rules. Whereas *agents* (computer systems capable of flexible, autonomous action to meet their design objectives^37^) refers to the biological entities being modelled and *environment* to the physical attributes of the space where the agents are being modelled, the *rules* are analogous to equations in the classical computational approach and contain the information protocols that regulate how agents interact with other agents as well as their environment. Furthermore, in agent-based models, the environment is not treated as a passive backdrop to the evolving agents, but as an able consort to these active entities – much like the stem cell *niche*^38^. Therefore, the environment (depending upon the system being analysed), influenced by the agent, alters simultaneously, in turn influencing the agents, thereby capturing *dynamis*m^33^: the signature of biology^39^. To find out more about agent-based modelling and the ontological relevance of agents to biological entities, especially the cell, the interested reader is directed to a review by Kaul & Ventikos^17^.

### Computational Platform

Computational simulations in this investigation were carried out in the agent-based platform *Flexible Large-scale Agent-based Modelling Environment* (FLAME). FLAME models agents as *communicating stream X-machines* (Supplementary Fig. S3), which allow the agents to interact with each other. FLAME uses a program known as the *Xparser* that parses the model XML definition into simulation program source code. Describing a system in FLAME involves identifying the agents, their environment, their functions, input and output messages of each function, as well as the set of variables (agents’ memory) that are accessed by the functions. The interested reader can find more information on FLAME (along with relevant downloadable files) at *www.flame.ac.uk/*.

### Rules

In this novel attempt to use rules, and the agent-based paradigm to capture (intramembranous) osteogenesis through cellular interactions, a very basic set of rules was derived by mining the literature on *in vitro* and *in vivo* osteogenesis manually. Rules assigned to the agents covered the entire spectrum of behaviours displayed by cells and, more specifically, pertained to proliferation, migration, differentiation, and apoptosis. Additionally, the four hypotheses of osteoblast transformation, explored in *Introduction*, were also converted into rules that governed bone deposition. The rules have been summarised in Table 2.

### Agents

The model used the major actors observed in the process of *in vitro* ossification, which include: fibroblast-like, colony-forming, precursor mesenchymal cells; pre-osteoblasts; osteoblasts; osteoid; mineralised osteoid; and osteocytes. To save computation time, other actors such as pre-osteoblastic osteoblasts or osteoid osteocytes were not considered without any effect on simulation outcome. All agents were modelled as non-deformable spheres, 20 μm in diameter. Although the platforms allows for the application of more complex and arbitrary 3D morphologies, the relatively simpler shapes were utilised to keep computational costs of running the model on a single desktop low.

While representing cells as agents was relatively straightforward – each cellular agent corresponded to one cell – computational representation of osteoid was slightly more challenging due to its heterogeneous nature. Osteoid is the unmineralised bone matrix synthesised by osteoblasts that consists of fibrous components (such as type I collagen) as well as ground substances (such as osteocalcin and chondroitin sulphate). However, as the set of behaviours explored in this investigation did not depend upon the heterogeneity of osteoid components, fibrous- and protein-based variations within osteoid were not considered, and an osteoid agent represented an arbitrary amount of homogeneous bone matrix, until it mineralised and turned into a separate agent (mineralised osteoid), as is the case physically.

### Physical Interactions

Forces between the aforementioned agents were resolved by implementing the *explicit* overlap detection and correction scheme. The term explicit implies that cellular displacement was determined based on the previous time step, instead of considering more than one previous time steps. The scheme detected and corrected any cellular overlap that occurred during mitosis and osteoid deposition. Any inter-agent overlap was corrected by applying a repulsive force proportional to the extent of the overlap. The underlying idea was to inhibit cell-cell overlap and, therefore, be able to model confluence dynamically, irrespective of the dimensions of the culture plate (a pervasive observation *in vitro*). The overlap occurred in the first place as FLAME executes the biological and physical rules serially, and not simultaneously, as is the case physically^40^. The scheme, adapted from the model employed by Adra *et al.* (2010)^40^, accounted for the contribution of mitosis and matrix apposition towards condensation growth, and has been mathematically described below.

For each iteration, the code checked whether any two neighbouring agents, *i* and *j*, are overlapping, and, in case of overlap, applying a repulsive force *F*_*c,ij*_, proportional to the overlap *O*_*ij*_, on the cell *i* by its neighbour *j*. The sub-script ‘*c*’ stands for the relevant coordinate under consideration (*x*, *y*, or *z*). The repulsive force was calculated as follows:


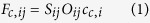


Considering the two agents have radii *r*_*i*_ and *r*_*j*_ (10 μm), and are centred at coordinates (*x*_*i*_, *y*_*i*_, *z*_*i*_) and (*x*_*j*_, *y*_*j*_, *z*_*j*_) respectively, the amount of overlap is shown in equation (2).


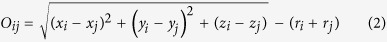


In equation (1), *s*_*ij*_ serves as an arbitrary ‘stiffness’ constant equal to the inverse of the separation between the two agents, refer to equation (3), and *c*_*c,i*_ represents the damping constant for each co-ordinate of agent *i*, refer to equations (4, 5, 6).


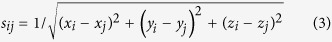



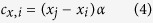



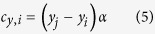



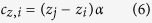


In equations (4, 5, 6), *α* represents the proportionality constant assigned based on the amount of agent overlap. In case there is no overlap (i.e. *O*_*ij*_ > 0), *α* = 0.06; but in case of overlap (i.e. *O*_*ij*_ > 0), *α* = 0.4. The new agent coordinates (*x*_*i,t*_, *y*_*i,t*_, *z*_*i,t*_) are determined by displacing the agent coordinates at the previous time step (*x*_*i,0*_, *y*_*i,0*_, *z*_*i,0*_) by (F_*x,ij*_, F_*y,ij*_, F_*z,ij*_) as shown in equations (7, 8, 9).


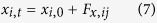



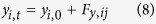



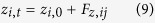


### Environment

Osteoid maturation and mineralisation *in vitro* is usually achieved by growing the cells to confluence in a culture dish followed by the addition of osteogenic factors^41^. As such, the environment used as the backdrop to the aforementioned agents constitutes a culture dish capable of supporting proliferation. The culture dish is assumed to be a 300 × 300 × 300 μm^3^. The size of the culture dish was limited to reduce computational costs. The studies quoted in this manuscript relied on static *in vitro* cultures, where the media was exchanged periodically; instead of applying dynamic perfusion. However, as supplement concentrations proved optimal in these investigations, resulting in normal mineralised condensations^20^,^22^,^42^ we assumed the virtual culture dish to contain optimal concentration of signalling molecules, growth factors, and hormones promoting ossification. The centre of this virtual culture dish served as the site of condensation in this model.

### Proliferation

*In vitro*, and indeed *in vivo*, only the precursor colony-forming mesenchymal and pre-osteoblastic cells possess the capacity to proliferate. The remaining cells, i.e. osteoblasts and osteocytes, do not. As such, in the model, only the precursor mesenchymal and pre-osteoblast agents were allowed to undergo mitosis. These agents divided roughly every 12 hours^43^, producing a daughter agent in a randomly chosen direction. The division of mesenchymal agents was limited to forming cells in the same two-dimensional plane; their pre-osteoblastic counterparts were allowed proliferation in the third dimension as well. The division of precursor mesenchymal cells, therefore, resulted in the formation of a monolayer of the initial colony-forming cells, whereas the proliferation of pre-osteoblastic agents led to condensation growth in the third dimension (in addition to growth in the same plane).

Proliferation of these two agents was ‘decentralised’, which means that it was contingent on the environment as a whole. For example, the precursor agents could proliferate until confluence, but once the culture dish became confluent, proliferation discontinued. However, in the event the precursor monolayer lost confluence, due to apoptosis or necrosis, the remaining cells could proliferate to reclaim the available space. This was achieved by adding the rule that each precursor agent could continue dividing until it achieves a maximum number of precursor neighbours. In computational terms, a non-overlapping agent in the vicinity, i.e. *O*_*ij*_ > 0 but ≤ 10 μm, constituted as a neighbour. Refer to equation (2) for the formula to determine *O*_*ij*_. The critical neighbour number was set to *four* for 2D proliferation (north, south, east, west) and six for 3D proliferation (top, bottom, north, south, east, west). The agents could establish neighbourhood with more than four neighbours, due to their vicinity, but they could not divide once the critical number of neighbours was achieved. Furthermore, pre-osteoblast proliferation in the third dimension, in addition to being limited by their neighbours, depended on spatial constraints as well.

### Migration

Migration is central to condensation formation and is only displayed by pre-osteoblasts *in vivo* (or, to be precise, cells that are no longer the colony-forming precursors but that have not differentiated into osteoblasts). The pre-osteoblasts migrate towards certain focal points to initiate condensation that are pre-determined, due to architectural constraints imposed by the environment. We, therefore, considered pre-osteoblast migration in the model. For visualisation purposes, the centre of the virtual culture dish served as the focal point towards which pre-osteoblasts migrated. In order to enable migration, each pre-osteoblast agent was assigned speed and directionality relative to the focal point. This was achieved by dividing the culture dish into four quadrants, which determined whether the cellular movement relative to the *x*- or *y*-axis was positive or negative. Assuming the coordinates of the focal point was represented by *ctr*_*x*_ and *ctr*_*y*_, and those of the migrating (*ith*) agent by *x*_*i*_ and *y*_*i*_ (bear in mind that migration only occurred in the same plane), the velocity, with respect to the two axes, was calculated as shown in Supplementary Table S2. Irrespective of their distance from the centre all mobile agents were assigned a constant speed of 0.2 μm/s.

Considering (*x*_*i,o*_, *y*_*i,o*_) represents agent coordinates for the *ith* agent at the previous time step and (*x*_*i,t*_, *y*_*i,t*_) at the next time step “*t*”, the new position of the migrating agent was calculated by:


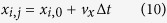



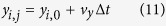


In equations (10 and 11), *v*_*x*_ and *v*_*y*_ represent the velocities in the *x* and *y* directions respectively (Supplementary Table S2), and Δ*t* the advance in iteration (Δ*t* = Δ*iteration* = 1 for all computations). At each iteration, the calculations, shown in Supplementary Table S2, equation (10), and equation (11), were repeated to determine agent velocity and displacement. Displacement was, thus, determined explicitly, which means that agent coordinates from only the previous time-step were considered in determining the coordinates for the new time step.

### Differentiation

A multitude of agents undergo differentiation within condensations. These include: precursor agents differentiating into pre-osteoblasts, pre-osteoblasts into osteoblasts, and osteoblasts, finally, terminally differentiating into osteocytes (refer to Table 2). The agents representing each of these cells possessed the capacity to differentiate. The precursor agents’ differentiation into pre-osteoblasts was stochastic. Only a limited number of precursor agents differentiated into pre-osteoblasts following confluence. Following condensation growth in the third dimension, only those pre-osteoblasts positioned well inside the condensation (height less than 80 μm and 20 μm away from the periphery) differentiated into osteoblasts. This constraint was imposed to account for the fact that cells exposed at the top of the condensation tend to be pre-osteoblastic in nature and only the ones ensconced within adopt the osteoblastic phenotype^20^. Furthermore, it is these pre-osteoblasts that are recruited following resorption to differentiate into osteoblasts and deposit bone. Finally, osteoblasts surrounded by mineralised osteoid (at least 6 mineralised neighbours covering the osteoblasts) terminally differentiate into osteocytes. Condensation height, which regulated the differentiation of pre-osteoblasts, was quantified by comparing the *z*-coordinates of the pre-osteoblast agents.

### Apoptosis

The osteoblasts within the condensation that that were unable to differentiate into osteocytes underwent programmed cell death, as has been reported elsewhere^6^. This, however, occurred stochastically and only after the nodule had acquired its maximum reported height (110 μm).

### Osteoid Deposition and Mineralisation

There are four hypotheses, as reported in *Introduction*, predicated on osteoblast polarity, which seek to explain the process of osteoid deposition and, thus, entrapment mechanism of osteoblasts. Osteoid could be deposited either in a random or a pre-determined direction. This feature is straightforward to incorporate into the model. The following rules for osteoid deposition were employed.

Hypothesis #1: Osteoblasts deposited osteoid in all directions. The direction at any given iteration was determined randomly. The osteoblast agent could alter its direction at any time during its lifetime.

Hypothesis #2: Each osteoblast, after its formation, randomly acquired a direction for osteoid deposition. However, unlike the mechanism represented in hypothesis #1, the osteoblast continued to deposit osteoid in that direction until its differentiation or apoptosis.

Hypothesis #3: Osteoblasts with similar z-coordinate, ± 10 μm apart, were considered part of the same layer, and deposited matrix in the same direction. These osteoblasts continued depositing matrix in the same direction until their differentiation or termination. In order to assign ‘layer’ based directionality, the space along condensation height was discretised into regions (10–30 μm, 30–50 μm, 50–70 μm, and 70–90 μm) each acquiring stochastically determined polarity. Therefore, the polarity eventually displayed by osteoblasts was acquired dynamically at run-time. This reflects the situation *in vivo*, where the osteogenic regions, by virtue of architectural constraints imposed by the environment, transmit to cells, localised within them, pre-determined polarities informed by the presence of nearby structures.

Hypothesis #4: In this case, osteoblast agents acquired polarity as in Hypothesis #3. Certain number (or population fraction) of osteoblasts, however, switched off their ability to produce matrix for a specific amount of time. A variety of switch-off periods from *indefinite* to *twenty four* hours were tested in this investigation.

Osteoid was only deposited by osteoblasts in the model. Furthermore, the model treated mineralisation stochastically – the ‘osteoid’ agent had to undergo two *if* loops before mineralising. Each osteoid agent represented a collection of deposited bone matrix (fibres and proteins). Osteoid heterogeneity was not considered to minimise computational costs.

### Computational Iterations and Sensitivity Analyses

A total of four models, incorporating the four hypotheses, were simulated in series. Each model was run for 5000 iterations, with one iteration equivalent roughly to 20 physical minutes. Each model was simulated eleven times in total across three different workstations. Hypothesis #3, identified as the fittest model, was used to test for sensitivity. Several parameters, related to osteoblast recruitment, osteoblast differentiation, and osteoblast vigour, were varied, which resulted in eight new simulations (n = 3). Hypothesis #4 was also tested for sensitivity. Parameters that were varied included the population fraction terminating osteoid synthesis, termination period, and condensation age at which the termination period was applied. Overall, thirteen parameters were varied (n = 3). Finally, the ability of the virtual condensations to recover from a compromised state was tested. This was achieved by reducing the condensation to a height of 40 μm and observing remodelling governed by each of the four hypotheses (n = 3). In order to further test the pathological nature of hypothesis #4, condensation remodelling by employing hypothesis #3 with abnormal osteoblast recruitment and osteoid deposition rate was observed (n = 3). The various runs have been summarised in Supplementary Tables S3-S6. Furthermore, refer to Supplementary Table S7 for a summary of our validation efforts.

### Statistical Analysis

Statistical analysis was carried out in SPSS. Osteocyte population count was tested using Kruskal-Wallis Test for non-normally distributed data and ANOVA for normally distributed data. The number of osteoblasts/osteocytes over each day and for each category was statistical tested using a two-way ANOVA with *time* and *case* as discrete outcomes. For post hoc correction of multiple comparisons of groups, two methods were used: Dunns and Bonferroni. Dunns was used when comparing the control only with the other groups, and Bonferroni was used for comparing all groups with each other. Cluster analysis was carried out using a mixture modelling statistical framework in SPSS^44^. The two-step cluster analysis method was used to test the number of clusters present in each iteration across the three dimensional multivariate space. The cluster algorithm worked by finding the split of the data that would best fit the data for the number of clusters specified. The best fitting model and, thus, the number of clusters that best fits the data were determined by calculating the Bayesian Information Criterion (BIC). The BIC test for model fitting uses both the statistical likelihood of the model as well as a penalisation for the number of parameters needed to determine the best fitting solution for the least amount of clusters. The cluster analysis was conducted on data sets, which contained the location of osteocytes for hypotheses #1, #2, and #3 (n = 11; 33 data sets were compared). The irregularity of osteocyte arrangement was based on both the visual assessment of osteocyte arrangement (refer to Supplementary Fig. S1) and the number of clusters found when cluster analysis was carried out for each simulation per hypothesis. If a hypothesis produced a consistent number of clusters, then this hypothesis was determined to be regular; if, however, the cluster analysis produced a more varied number of clusters for each simulation of the same hypothesis then this arrangement was deemed irregular (refer to Supplementary Table S1). Overall, data from more than 125 computational iterations were analysed and presented in this manuscript.

## Additional Information

**How to cite this article**: Kaul, H. *et al.* Synergistic activity of polarised osteoblasts inside condensations cause their differentiation. *Sci. Rep.* **5**, 11838; doi: 10.1038/srep11838 (2015).

## Supplementary Material

Supplementary Information

## Figures and Tables

**Figure 1:**
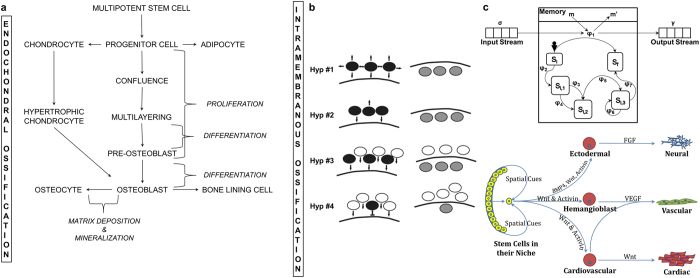
Osteoblast differentiation pathway, the underlying mechanisms, and the ontological relevance of ‘agents’ to cells. **(a)** represents the list of events and differentiation pathways that progenitor cells undergo before transitioning into osteoblasts and, eventually, osteocytes. Of relevance to this investigation are events displayed in the centre, in which bone forms through intramembranous ossification. In endochondral ossification, the alternative pathway, hypertrophic chondrocytes have been indicated to differentiate into osteoblast-like cells. The figure was adapted from^23^. In **(b)**, the possible ways (hypotheses #**1–4**) of matrix deposition by osteoblasts, as presented in^6^, are shown. The left column represent the situation before osteoblasts are trapped within the matrix. The arrows represent the direction of matrix deposition by osteoblasts. Black shaded cells represent the cells that will be entrapped within the matrix and the solid line represents the bone surface. The grey shaded cells in the right column indicate former osteoblasts that turn into osteocytes. **(c)** Agents are computer programs that are capable of detecting local information and initiate decision making based on a set of rule-set attributed to them at discrete time steps. In that sense, they act very much like a biological cell. In this frame, a parallel between an agent and a cell is shown. Based on the incoming cue (signalling molecule, architectural constraint, mechanical conditioning, etc.) both the agent and the cell end up changing their ‘state’ (to chemotaxis or differentiation, etc.), producing an output signal (autocrine or paracrine), and updating their memory (i.e. the new differentiated state, etc.). ***(b)** was reproduced with kind permission from* Ref. 6 *© (2005) John Wiley and Sons and **(c)** was reproduced with kind permission from Ref. 17 © (2013) Oxford University Press*.

**Table 1:** A summary of the sensitivity analysis.

Case	Parameter	Variation	Original	Result	Maturation	Average Osteocytes
h3	Hypothesis #3	Original	Original	Osteocytes observed on day 9 pc; Osteocyte number roughly equal to 86 ± 5	day 9	86 ± 5
S1	Pre-osteoblast proliferation	12 ± 3 hours	12 hours	Delay observed in condensation formation (day 10pc); number of osteocytes roughly the same (83 ± 3)	day 10	83 ± 3
S2	Pre-osteoblast proliferation	12 ± 1 hours	12 hours	Similar to the original in terms of condensation formation rate (day 9pc) and number of osteocytes formed (84 ± 5)	day 9	84 ± 5
S3	Pre-osteoblast proliferation	8 hours	12 hours	Earliest condensation and osteocyte formation observed (day 7pc); number of osteocytes relatively unaffected (96 ± 5)	day 7	96 ± 5
S4	Matrix surrounding Osteoblasts	4 neighbours	6 neighbours	Similar in terms of condensation formation (day 9pc); but relatively higher number of osteocytes observed (101 ± 3)	day 9	101 ± 3
S5	Matrix deposition rate	6 hours	18 hours	Early condensation formation observed (day 8pc); relatively similar number of osteocytes (76 ± 1)	day 8	76 ± 1
S6	Matrix deposition rate	18 ± 3 hours	18 hours	Similar to the original in terms of condensation formation rate (day 9pc) but fewer osteocytes (53 ± 5)	day 9	53 ± 5
S7	Matrix deposition rate	1 hour	18 hours	Condensation formation earliest (day 7pc); but very few osteocytes observed (30 ± 0)	day 7	30 ± 0
S8	Matrix deposition rate/Pre-osteoblast proliferation	1 hour/18 hours	18 hours/ 12 hours	Lack of enough osteoblasts (37 ± 3) as well as extremely delayed condensation formation (day 11 pc)	day 11	37 ± 3

The table lists the various simulations conducted to test model’s sensitivity to various parameters governing condensation development. It also details the variables that were altered and summarises the results from each test case (n = 3). In order to provide context to the analysis, the time of condensation maturation and the average osteocyte population observed towards the end of 12^th^ day p.c. is also provided.

**Table 2:** Agents, rules, and fates.

Activity	Agent(s)	Rule		Agent Fate	Visualisation
Proliferation	Precursor	Initiates at the very beginning		Agent divides to produce more daughter agents	Blue Sphere
		The agent divides every 12 hours producing a daughter agent in a randomly chosen direction			
		The agent cannot divide in the third dimension			
		Stops once confluence has been achieved			
	Pre-osteoblasts	Initiates after confluence		Agent division promotes condensation development	
		The agent divides every 12 hours producing a daughter agent in a randomly chosen direction. Time was varied while testing for sensitivity to 12 ± 3, 12 ± 1, and 8 hours			
		The agent can divide in the third dimension			
		Proliferation continues until the condensation height reaches 90 μm			
Migration	Pre-osteoblasts	Starts after confluence		Agent migration towards the skeletogenic site helps initiate condensation development	Blue (and Orange in Fig. 2b) Sphere
		Randomly chosen agents migrate towards the site of skeletogenesis			
		Stops after pre-osteoblasts have arrived at the skeletogenic site and started proliferating			
Apoptosis	Osteoblasts	Initiates once the condensation height reaches 110 μm		Agent dies and plays no further role in the simulation. The space becomes available to other agents	Green Sphere disappears
		The agent will enter apoptotic cycle if it has not differentiated into an osteocyte at the aforementioned stage			
Differentiation	Precursor	Initiates after confluence		Agents differentiate into pre-osteoblasts that migrate to the skeletogenic site and initiate condensation development	Blue Sphere remains Blue (except in Fig. 2b where they were changed to orange to better visualise migration)
		Randomly chosen agents differentiate into pre-osteoblasts			
		Stops once a certain randomly chosen number of agents have differentiated			
	Pre-osteoblasts	Initiates once the condensation height reaches 50 μm		Agent differentiates into an osteoblast that deposit osteoid to further develop the condensation	Blue Sphere turns Green
		The agent must be within the condensation (as such this feature constantly evolves with the condensation), OR			
		The agent is in contact with more than four Osteoblasts			
		Pre-osteoblasts turn into Osteoblasts			
		Osteoblasts can turn back into pre-osteoblasts if they are at the periphery of the condensation			
	Osteoblasts	Initiates once the condensation height reaches 100 μm		Agent differentiates into an osteocyte	Green Sphere turns Black
		The agent, if embedded within or surrounded by calcified matrix (6 matrix agents), differentiates into Osteocyte. In sensitivity analysis, the number of neighbours was decreased to 4			
		Continues until all osteoblasts have either differentiated or apoptosed			
	Matrix Calcification	Initiates once the condensation height reaches 100 μm		Calcified matrix buries osteoblasts that assist their differentiation into osteocytes	Red Cube turns Grey
		All matrix agents calcify slowly			
Matrix Deposition	Osteoblasts	Initiates once the condensation height reaches 70 μm		Agent deposits matrix that further leads to condensation development. The agent is then entrapped in the calcified form of the matrix to undergo differentiation into an osteocyte	Green Sphere produces Red Cubes
		H#1	Agent deposits matrix randomly in any of the three-dimensions throughout the simulation		
		H#2	Agent, once formed, acquires polarity randomly and continues to deposit matrix in that direction for the entirety of the simulation		
		H#3	Polarity is treated as a property of an agent layer		
			Agents within 20 μ m of each other were treated as belonging to the same layer		
			Agents acquired polarity once formed (with condensation height exceeding 50 μm)		
		H#4	Same as H#3		
			Within a certain duration, some randomly chosen agents, less than 2% of the entire population, turn off their genes to secrete matrix		
		Matrix secretion ceases once the condensation height exceeds 110 μm			

The table lists the agents utilised in this investigation as well as the rules that governed their behaviour. The table also features the agent fate eventuating from the agents following the rules as well as the manner in which this was visualised in the figures presented in this paper.
