# Supplementary material for: Synergistic activity of polarised osteoblasts inside condensations cause their differentiation
Source: Sci Rep. 2015 Jul 6;5:11838. doi: 10.1038/srep11838 (PMC4491713; doi:10.1038/srep11838)
Supplement: Supplementary Information [file srep11838-s1.doc]

**Supplementary Material**

**Synergistic activity of polarised osteoblasts inside condensations cause their differentiation**

**Running Title: Polarised osteoblasts form osteocytes**

Himanshu Kaul^1,2^*†, Brian K. Hall^3^, Chris Newby^4^, Yiannis Ventikos^5^*

**Affiliations**

^1^Department of Engineering Science, University of Oxford, Oxford, OX1 3PJ, UK.

^2^Kroto Research Institute, Department of Computer Science, University of Sheffield, Sheffield, S3 7HQ, UK.

^3^Department of Biology, Dalhousie University, Halifax, Nova Scotia, B3H 4R2, Canada.

^4^Department of Infection, Immunity, and Inflammation, University of Leicester, Leicester, LE1 9HN, UK.

^5^Department of Mechanical Engineering, University College London, London, WC1E 7JE, UK.

*Correspondence to: h.kaul@sheffield.ac.uk and y.ventikos@ucl.ac.uk

†Current Address: Kroto Research Institute, Department of Computer Science, University of Sheffield, Sheffield, S3 7HQ, UK.

**Supplementary Figures**

**Supplementary Figure S**1

| **Time,**  **days p.c.** | **Hypothesis #1 Hypothesis #2 Hypothesis #3 Hypothesis #4** |
| --- | --- |
| **7**  **9**  **11**  **12**  **14**  **16** | 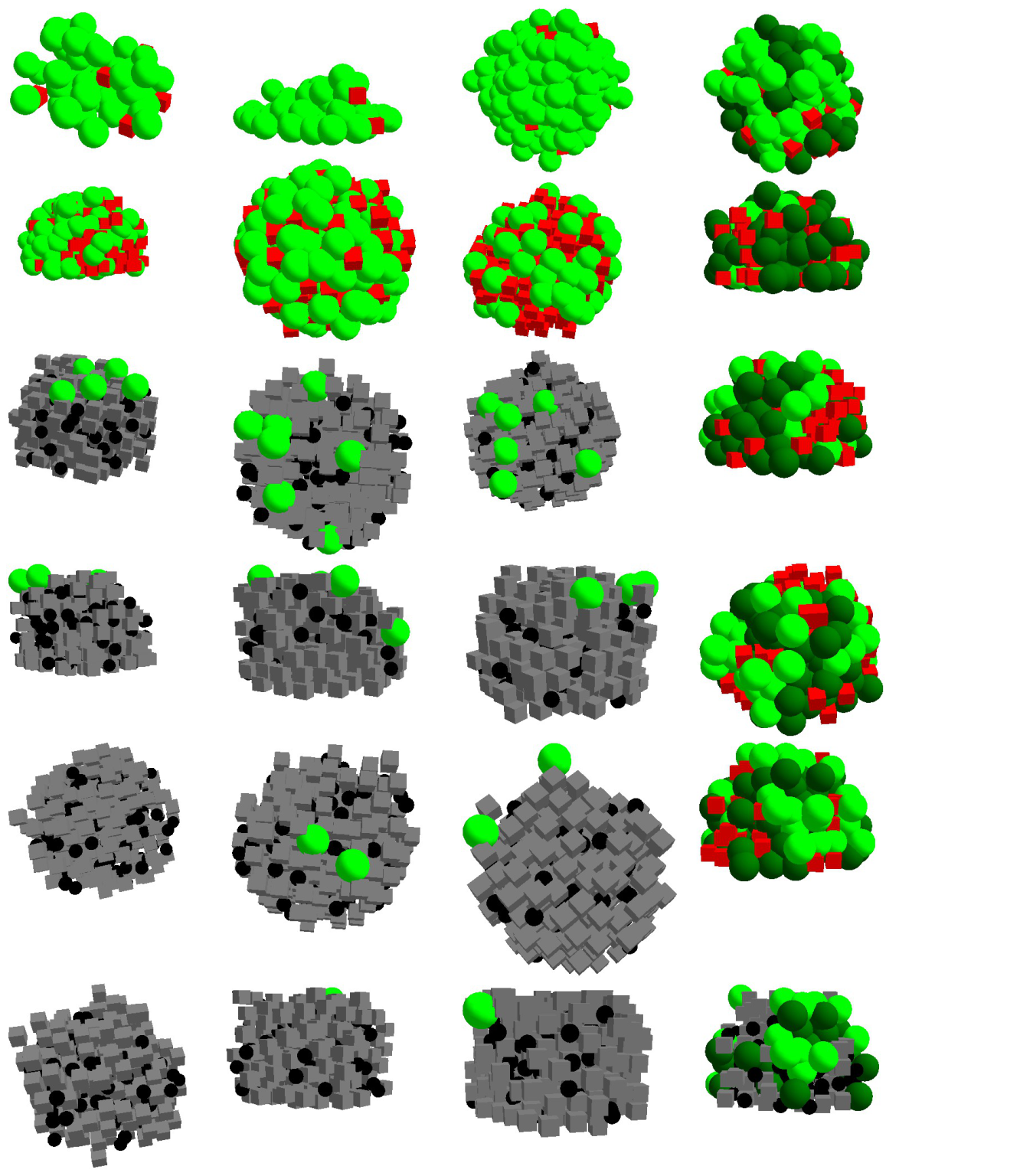 |

**Supplementary Figure S1. Osteoblastic transformation into osteocytes without Mesenchymal cells.** The figure captures the spatiotemporal development of the condensation in terms of matrix deposition, its mineralisation, and the terminal differentiation of osteoblasts into osteocytes. Frames presented in this figure were captured from various angles. A couple of conclusions can be easily drawn from the frames presented in this figure: condensation growing per hypothesis #4 fails to achieve the requisite condensation size and therefore osteoblast differentiation; and matrix deposition occurs in the most ordered manner governed by hypothesis #3.

**Supplementary Figure S2**


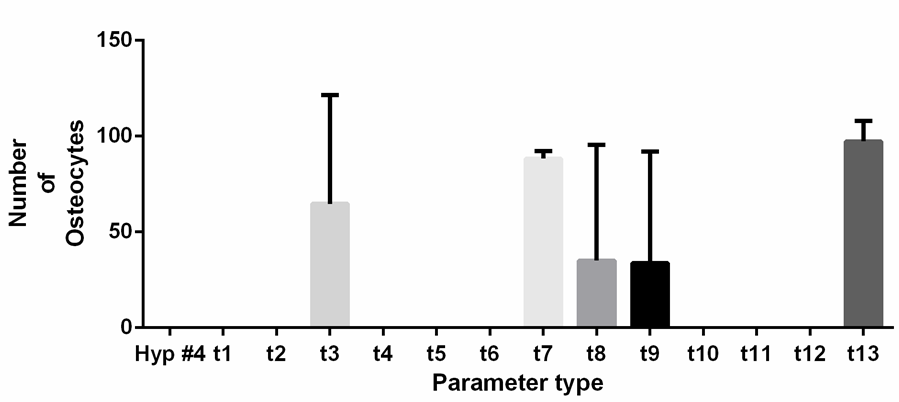


**Supplementary Figure S2. Sensitivity analysis of hypothesis #4 as the mechanism governing condensation development.** The graph represents the number of osteocytes obtained when parameters regarding osteoid ‘switch-off’ were altered. Parameters altered included the percentage frequency of osteoblasts switching off their osteoid deposition genes (30%, 10%, 2%), the amount of time during which this decision was made (indefinite, 2 days, 1 day), and the moment during simulation when this behaviour was allowed (early vs late). Osteocytes were only observed when the population switching-off osteoid deposition was reduced to 2%, the period to 1 day, and when this condition was imposed quite early during simulation. This indicated that normal osteocyte population can be observed as hypothesis #4 approaches hypothesis #3. Please refer to *Materials and Methods* for details regarding the analysis.

**Supplementary Figure S3**


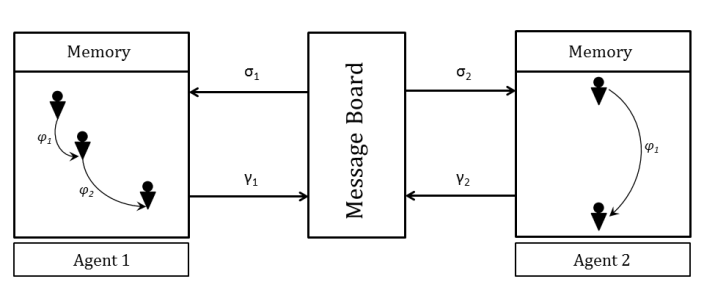


**Supplementary Figure S3. X-machine communication.** The figure demonstrates message exchange between agents via a message board that maintains a database of all messages sent by agents. *Reproduced with kind permission from Ref. 1 © (2013) Oxford University Press*.

**Supplementary Tables**

**Supplementary Table S**1

| **Number of clusters detected** | | | |
| --- | --- | --- | --- |
| **Iteration #** | **Hypothesis #1** | **Hypothesis #2** | **Hypothesis #3** |
| **1** | 2 | 2 | 2 |
| **2** | 2 | 3 | 2 |
| **3** | 3 | 2 | 2 |
| **4** | 2 | 1 | 2 |
| **5** | 2 | 2 | 2 |
| **6** | 3 | 2 | 3 |
| **7** | 2 | 2 | 2 |
| **8** | 3 | 3 | 2 |
| **9** | 2 | 2 | 2 |
| **10** | 3 | 2 | 2 |
| **11** | 3 | 2 | 3 |

**Supplementary Table S1. Number of osteocyte clusters per hypothesis.** The table lists the number of osteocyte clusters observed for simulations conducted employing hypotheses #1-3. Data from 11 simulations conducted on three different computers (5-3-3) are shown. Inconsistency in cluster formation when employing hypothesis #2 can be observed by the variation in the number of clusters that can form (three as opposed to two for hypothesis #1). Osteocytes were not observed in simulations employing hypothesis #4 during normal time frame and, therefore, were not included in this analysis.

**Supplementary Table S2**

| ***ctr_x_ – x_i_*** | ***ctr_y_ – y_i_*** | ***Velocity_x (µm/s)*** | ***Velocity_y (µm/s)*** |
| --- | --- | --- | --- |
| *+ve* | *+ve* | *0.2* | *0.2* |
| *+ve* | *–ve* | *0.2* | *-0.2* |
| *–ve* | *+ve* | *-0.2* | *0.2* |
| *–ve* | *–ve* | *-0.2* | *-0.2* |

**Supplementary Table S2. Pre-osteoblast migration speed and direction.** The table shows the manner in which pre-osteoblasts were assigned speed and direction. Only a selected number of precursor cells differentiated into pre-osteoblasts, which depending on their coordinates moved towards the focal point of the culture dish chosen for condensation. Based on their direction from the condensation site, they were assigned a particular velocity. For example, if an agent was on top left of the centre of the culture dish, it was assigned a velocity of 0.2 µm/s to move right and -0.2 µm/s to move down.

**Supplementary Table S3**

| **Case** | **Osteoblast Polarity** | **Osteoid Switch-off** | **Runs (n)** | **Computer #** |
| --- | --- | --- | --- | --- |
| **hypothesis #1** | Apolar | NA | 11 | 1, 2, 3 |
| **hypothesis #2** | Stochastic | NA | 11 | 1, 2, 3 |
| **hypothesis #3** | Layered | NA | 11 | 1, 2, 3 |
| **hypothesis #4** | Layered | Yes | 11 | 1, 2, 3 |

**Supplementary Table S3. Hypothesis testing approach.** The table lists the mechanisms investigated in this study and the manner in which each treated osteoblast polarity. Each hypothesis was simulated 11 times on three different workstations to ensure the code was insensitive to stochastic variables within the code as well as dissimilar random number generation between different workstations.

**Supplementary Table S4**

| **Case** | **Parameter** | **Classification** | **Variation** | **Original** | **Runs (n)** |
| --- | --- | --- | --- | --- | --- |
| **h3** | Original | h3 | Original | Original | 3 |
| **S1** | Pre-osteoblast proliferation | Osteoblast Recruitment | 12±3 hours | 12 hours | 3 |
| **S2** | Pre-osteoblast proliferation |  | 12±1 hours | 12 hours | 3 |
| **S3** | Pre-osteoblast proliferation |  | 8 hours | 12 hours | 3 |
| **S4** | Matrix surrounding Osteoblasts | Differentiation | 4 neighbours | 6 neighbours | 3 |
| **S5** | Matrix deposition rate | Osteoid Synthesis | 6 hours | 18 hours | 3 |
| **S6** | Matrix deposition rate |  | 18±3 hours | 18 hours | 3 |
| **S7** | Matrix deposition rate |  | 1 hour | 18 hours | 3 |
| **S8** | Matrix deposition rate/Pre-osteoblast proliferation | Osteoblast Recruitment & Osteoid Synthesis | 1 hour/18 hours | 18 hours/12 hours | 3 |

**Supplementary Table S4. Sensitivity testing on hypothesis #3.** The table lists the various parameters altered as part of the sensitivity analysis to determine the features the model was sensitive to. Parameters varied could be divided into four categories listed in the table. Each new model (s1 – s8) was simulated three times.

**Supplementary Table S5**

| **Case** | **Population Fraction** | **Switch-off Period** | **Switch-off Time (days p.c.)** | **Computer #** | **Runs (n)** |
| --- | --- | --- | --- | --- | --- |
| **h4** | 2% | indefinite | 5.75 | 1 | 3 |
| **t1** | 2% | 2 days | 5.75 | 1 | 3 |
| **t2** | 10% | 1 day | 5.75 | 1 | 3 |
| **t3** | 2% | 1 day | 5.75 | 1 | 3 |
| **t4** | 30% | 1 day | 5.75 | 1 | 3 |
| **t5** | 10% | indefinite | 5.75 | 1 | 3 |
| **t6** | 2% | indefinite | 5.75 | 1 | 3 |
| **t7** | 2% | 1 day | 4.375 | 1 | 3 |
| **t8** | 2% | 1 day | 6.875 | 1 | 3 |
| **t9** | 2% | 1 day | 7.5 | 1 | 3 |
| **t10** | 10% | 2 days | 5.75 | 2 | 3 |
| **t11** | 2% | 2 days | 5.75 | 2 | 3 |
| **t12** | 10% | 1 day | 5.75 | 2 | 3 |
| **t13** | 2% | 1 day | 5.75 | 2 | 3 |

**Supplementary Table S5. Sensitivity testing on hypothesis #4.** The table lists the changes made to the model simulating osteoid deposition based on hypothesis #4. This sensitivity analysis was conducted to determine the parameters hypothesis #4 was most sensitive to. Three basic parameters were tested: ‘population fraction’ turning of osteoid deposition; the duration of time during which osteoid deposition was ‘switched-off’; and the time when this switch-off came into effect. In order to ensure code’s insensitivity to inter-computer and intra-code stochasticity, some of the models were simulated on another computer. Each model was simulated three times.

**Supplementary Table S6**

| **Case** | **Title** | **Pre-osteoblast Proliferation** | **Osteoid Deposition** | **Runs (n)** |
| --- | --- | --- | --- | --- |
| **h1** | Hypothesis #1 | 12 hours | 18 hours | 3 |
| **h2** | Hypothesis #2 | 12 hours | 18 hours | 3 |
| **h3** | Hypothesis #3 | 12 hours | 18 hours | 3 |
| **h4** | Hypothesis #4 | 12 hours | 18 hours | 3 |
| **r1** | Matrix Overproduction | 12 hours | 1 hour | 3 |
| **r2** | Low Osteoblast Vigour | 18 hours | 27 hours | 3 |

**Supplementary Table S6. Remodelling following the ‘resorption’ challenge.** The table lists the variables utilised to observe osteogenic remodelling following ‘resorption’ and necrosis of the mineralised osteoid and osteocytic/pre-osteoblastic cell populations, respectively. Remodelling was investigated using the original four hypotheses (h1 – h4), as well as using (abnormal) hypothesis #3, which was modified to represent the pathological states of (r1) matrix overproduction and (r2) low osteoblast vigour (i.e. low osteoblast recruitment and delayed matrix deposition). Each model was simulated three times.

**Supplementary Table** S7

| **Model Output** | **Output** | **Comment** | **Validation against** |
| --- | --- | --- | --- |
| **Nodule maturation** | ~9 days post-confluence | This matches the range observed in investigations conducted *in vitro* | 2-6 |
| **Nodule Structure** | Arrangement of cells within the nodule | Osteoblast-like cells can be observed at the fringes of the nodule. The core contains mineralised osteoid and osteocytes | 2-7 |
| **Osteocytes within the nodule** | ~85 | Nodule cellularity has been quantified as 100 cells/nodule. Our model produces statistically significant number of cells, as can be seen in our analyses where ‘normal’ condensations can contain 75 – 104 osteocytes. Furthermore, given there are 10x as many osteocytes as osteoblasts, the total number of osteocytes within a nodule can be assumed to be ≈90. Again, this is statistically similar to our output. | 2,8 |
| **Osteocyte proportion within the nodule** | Highest | Osteocytes form the bulk of the cells in mature nodules | 5,9 |
| **Impact of osteoblast polarity on osteocyte arrangement within the nodule** | Both irregular (hypothesis #2) and regular (hypothesis #3) arrangement observed | Ferretti et al. report that osteoblasts that act synchronously result in osteocytes that are regularly arranged (as is the case with hypothesis #3) and the osteoblasts that secrete matrix in one direction through their life-time after acquiring that orientation result in osteocytes that are organised irregularly (as is the case with hypothesis #2) | 10 |
| **Impact of polarity on osteoblast ‘burial’** | Self-burial as well as burial due to activity of neighbouring osteoblasts | Osteoblasts that act synchronously bury their neighbouring osteoblasts (hypothesis #3), whereas osteoblasts that deposit osteoid in one direction throughout their life (and do not alter their orientation after acquiring it initially) end up undergoing self-burial (hypothesis #2) | 10 |
| **Factors essential for normal nodule formation** | Osteoblast recruitment and osteoid synthesis | Our statistical analyses reveal that normal condensation development is predicated on normal functioning of both osteoblast recruitment (captured within our model of in vitro osteogenesis by pre-osteoblast proliferation) and osteoid deposition. The only hypothesis where one acts abnormally (osteoid production in hypothesis #4) fails to show abnormal development. Furthermore, our analysis with the ‘remodelling following resorption’ experiment also reveal that both of these features must act normally to result in normal nodular structure | 2,6,9,11 |
| **Osteogenic Template** | Cellular proliferation -> cell differentiation -> condensation -> osteoid deposition -> terminal differentiation (including mineralisation) | Our model cleanly captures the events as proposed by Hall and Miyake, and those observed empirically *in vitro* | 12,13 |

**Supplementary Table S7. Model Validation.** The table lists the various *explicit* and *semi-explicit* means to validate the model as well as the underlying rule-sets. The table also lists the *in vitro* and *in vivo* investigations that our model outputs were compared with.

**SUPPLEMENTARY MATERIAL REFERENCES**

1. Kaul, H. & Ventikos, Y. Investigating biocomplexity through the agent-based paradigm. *Brief Bioinform*, doi:10.1093/bib/bbt077 (2013).
2. Beresford, J. N., Graves, S. E. & Smoothy, C. A. Formation of mineralized nodules by bone derived cells in vitro – a model of bone formation? *Am. J. Med. Genet.* **45**, 163-178, (1993).
3. Lian, J. B. & Stein, G. S. Development of the osteoblast phenotype: molecular mechanisms mediating osteoblast growth and differentiation. *Iowa Orthop. J.* **15**, 118-140 (1995).
4. Bellows, C. G., Aubin, J. E., Heersche, J. N. M. & Antosz, M. E. Mineralized bone nodules formed in vitro from enzymatically released rat calvaria cell populations. *Calcif. Tissue Int.* **38**, 143-154 (1986).
5. Franz-Odendaal, T. A., Hall, B. K. & Witten, P. E. Buried alive: How osteoblasts become osteocytes. *Dev. Dyn.* **235**, 176-190 (2006).
6. Bhargava, U., Barlev, M., Bellows, C. G. & Aubin, J. E. Ultrastructural analysis of bone nodules formed in vitro by isolated fetal-rat calvaria cells. *Bone* **9**, 155-163 (1988).
7. Hancox, N. M. & Boothroyd, B. Electron microscopy of the early stages of osteogenesis. *Clin Orthop Relat Res* **40**, 153-161 (1965).
8. Bellows, C. G. & Aubin, J. E. Determination of numbers of osteoprogenitors present in isolated fetal rat calvaria cells in vitro. *Dev. Biol.* **133**, 8-13 (1989).
9. Parfitt, A. M. Bone-forming cells in clinical conditions. *Bone Vol. 1: the osteoblast and osteocyte* [Hall, B.K. (ed.)] [Ch. 9, 351-429] (Telford Press and CRC Press, Florida, 1990).
10. Ferretti, M., Palumbo, C., Contri, M. & Marotti, G. Static and dynamic osteogenesis: Two different types of bone formation. *Anat. Embryol. (Berl.)* **206**, 21-29 (2002).
11. Jabalee, J., Hillier, S. & Franz-Odendaal, T. A. An investigation of cellular dynamics during the development of intramembranous bones: the scleral ossicles. *J. Anat.* **223**, 311-320 (2013).
12. Hall, B. K. & Miyake, T. Divide, accumulate, differentiate: Cell condensation in skeletal development revisited. *Int. J. Dev. Biol.* **39**, 881-893 (1995).
13. Hall, B. K. & Miyake, T. All for one and one for all: condensations and the initiation of skeletal development. *Bioessays* **22**, 138-147 (2000).
